# Temporal trends of β-haemolytic streptococcal osteoarticular infections in western Norway

**DOI:** 10.1186/s12879-016-1874-7

**Published:** 2016-10-04

**Authors:** Oddvar Oppegaard, Steinar Skrede, Haima Mylvaganam, Bård Reiakvam Kittang

**Affiliations:** 1 Department of Medicine, Haukeland University Hospital, 5021 Bergen, Norway; 2 Department of Clinical Science, University of Bergen, Bergen, Norway; 3 Department of Microbiology, Haukeland University Hospital, Bergen, Norway; 4 Department of Medicine, Haraldsplass Deaconess Hospital, Bergen, Norway

**Keywords:** *Streptococcus dysgalactiae*, S*treptococcus pyogenes*, *Streptococcus agalactiae*, Osteoarticular infection, Septic arthritis, Epidemiology

## Abstract

**Background:**

Beta-haemolytic streptococci are important contributors to the global burden of osteoarticular infections (OAI). Knowledge on the disease traits specific for streptococcal OAI, however, remains scarce. We wished to explore temporal trends of OAI caused by Group A Streptococci (GAS), Group B Streptococci (GBS) and Group C and G Streptococci (GCGS), and furthermore, to describe the associated host and pathogen characteristics.

**Methods:**

All cases of microbiologically verified β-haemolytic streptococcal OAI in Health Region Bergen, Norway, in the period 1999–2013 were retrospectively identified. Clinical data were extracted from medical records. Microbial isolates were submitted to antibiotic susceptibility testing and molecular typing.

**Results:**

A total of 24 GAS, 45 GBS and 42 GCGS acute OAI were identified. The cumulative incidence of GCGS OAI, but not GAS or GBS OAI, increased significantly from the first to the last 5-year period (IRR 5.7, *p* = 0.0003), with the annual incidence peaking at 1.9/100 000 in 2013. GAS OAI generally produced the most acute and severe clinical presentation, whereas GBS and GCGS predominantly affected the elderly, and were significantly associated with the presence of host risk factors of systemic and focal origin, respectively.

**Conclusions:**

We found a significantly increasing incidence of GCGS OAI, likely related to the presence of host susceptibility factors, including prosthetic material and pre-existing joint disease. With an increasing application of therapeutic and diagnostic bone and joint procedures, the rising trend of OAI caused by GCGS is likely to continue. Sustained epidemiological attentiveness to GCGS seems warranted.

## Background

Osteoarticular Infections (OAI) cause significant morbidity worldwide, and the incidence is increasing [1–4]. This likely reflects both a higher burden of comorbidity in the population, and an increased performance of surgical bone and joint procedures [1, 2].

Streptococci are consistently second only to staphylococci as causative agents of OAI, representing approximately 15–20 % of the cases [4–6]. However, knowledge on clinical and microbial characteristics specific for streptococcal OAI is scarce, and mostly available from small case series published decades ago [7]. The few retrospective cohort studies published revealed a predominance of β-haemolytic streptococci, with group A Streptococci (GAS) and group B streptococci (GBS) comprising the majority [8–10]. Furthermore, species-associated clinical features were suggested; GBS mainly affecting the multi-morbid and elderly, and GAS producing more severe clinical manifestations. However, the number of included cases in these studies was generally too small to permit statistical analyses or evaluate temporal trends.

*Streptococcus dysgalactiae* subspecies *equisimilis* (SDSE), a β-haemolytic streptococcus predominantly possessing Lancefield group C or G antigen (GCS and GGS), has recently emerged as an important pathogen, increasingly associated with invasive disease [11]. We wished to explore if this rising trend was reflected in the rates of osteoarticular GCS and GGS-infections, and to describe the host and pathogen characteristics of these infections in comparison to OAI caused by GAS and GBS.

## Methods

### Study setting

Health Region Bergen, Western Norway, comprises the tertiary care hospital Haukeland University Hospital, and the two secondary care hospitals Haraldsplass Deaconess Hospital and Voss Hospital. The population in the catchment area increased from 360 796 to 427 486 inhabitants during the study period. The epidemiology of invasive β-haemolytic streptococcal disease within this region in the period 1999–2013 has previously been described [11]. Cases conforming to the OAI case definition were included, and demographic and clinical information was abstracted from medical records.

### Definitions

Osteoarticular infections comprised Native Joint Infections (NJI), defined as Newman group A and B [12], Prosthetic Joint Infection (PJI), identified and classified according to IDSA guidelines [13], and Acute Osteomyelitis (AOM), inferred from a radiologically verified bone-infection in conjunction with a positive blood culture or bone biopsy obtained though intact skin. AOM included both peripheral and vertebral osteomyelitis. Cases with chronic osteomyelitis (symptom duration >4 weeks) or concurrent endocarditis, were excluded. Repeatedly positive cultures with the same organism identified within 30 days of initial isolation were considered to be from a single episode. Relapse was defined as a clinical deterioration after cessation of antibiotic treatment, of verified β-hamolytic streptococcal or unidentified microbiological aetiology, resulting in a new medical or surgical intervention. Referrals from other hospitals and patients with a registered permanent residence address outside the catchment area at the time of admission were excluded. Systemic inflammatory response syndrome (SIRS) and Streptococcal toxic shock syndrome (STSS) were defined as previously described [14, 15]. Sequelae were defined as presence of arthrosis, chronic joint pain, reduced joint motility, amputation or chronic infection treated with suppressive antibacterial therapy.

### Bacterial identification, typing and antibiotic susceptibility

All isolates displayed large colony size (>0.5 mm in diameter after 24 h) and a β-haemolytic reaction on 5 % sheep blood agar. Serogroup specificity was determined using a rapid agglutination test (Oxoid Streptococcal Grouping Kit, Hampshire, UK). As β-haemolytic GCS and GGS causing human infections most frequently belong to the species SDSE, and the number of GCS was very low, a decision was made to treat them as one group (GCGS) for statistical purposes. Nonculturable bacteria were identified by *16 s rDNA*-PCR amplification from the sample material and subsequent sequencing, using primers and protocol as described previously [16]. Antimicrobial susceptibility testing of the invasive isolates had been performed according to Norwegian guidelines (http://www.unn.no/fag-og-forskning/arbeidsgruppen-for-antibiotikasporsmal-afa). However, routine testing for erythromycin-susceptibility and inducible clindamycin resistance was not performed prior to 2004. *emm*-typing of GAS, GCS and GGS was performed as described elsewhere [17]. GBS were serotyped by ImmuLex™ Strep-B latex-agglutination (SSI Diagnostica, Denmark).

### Statistics

Data were analysed using SPSS PASW STATISTICS (IBM SPSS Statistics for Windows, Version 21.0. Armonk, NY: IBM Corp). Categorical data were analysed using Chi square test or Fisher’s exact test as appropriate. Non-parametric data were analysed using the Mann-Whitney *U*-test. For Tables 1 and 2 the *p*-values were adjusted for multiple comparisons ad modum Holm-Bonferroni. Population-data for the catchment area was obtained from Statistics Norway (http://www.ssb.no). Incidence rates were age and sex-adjusted to the 2001 Norwegian standard population. Time trends were evaluated by dividing the data into an early cohort (1999–2003) and a late cohort (2009–2013), and calculating mean incidence rate ratios (IRR) and a *p*-value. A two-sided *p*-value ≤0.05 was considered statistically significant.

**Table 1:** Demographics, comorbidity/risk factors and disease manifestations

	GAS *n* = 24		GBS *n* = 45		GCGS *n* = 40 ^a^		GAS vs GBS	GAS vs GCGS	GBS vs GCGS
Demographics							Adjusted *p*-values ^*^		
Male sex	15	63 %	21	47 %	25	63 %	0.62	1.0	0.95
Age Median (IQR)	48	(8–72)	70	(58–80)	69	(49–81)	**0.007**	**0.049**	1.0
Disease manifestation									
AOM	5	21 %	11	24 %	4	10 %	1.0	1.0	0.6
Vertebral AOM	0		7		3				
Peripheral AOM	5		4		1				
PJI	5	21 %	22	49 %	15	38 %	0.148	1.0	1.0
Early PJI	2		6		2				
Delayed PJI	3		6		5				
Late PJI	0		10		8				
NJI	14	58 %	12	27 %	21	53 %	**0.049**	1.0	0.11
Affected joint									
Hip	4	21 %	15	44 %	9	25 %			
Knee	8	42 %	8	24 %	15	42 %			
Ankle	2	11 %	1	3 %	3	8 %			
Shoulder	0	0 %	1	3 %	4	11 %			
Other	7	37 %	6	18 %	11	31 %			
Multiple joints	0	0 %	5	15 %	1	3 %			
Microbiological cultures									
Blood	14	58 %	25	56 %	13	33 %			
Synovial Fluid	14	58 %	11	24 %	23	58 %			
Tissue	1	4 %	12	27 %	8	20 %			
Polymicrobial ^b^	3	13 %	2	4 %	4	10 %			
Comorbidity and risk factors									
Systemic risk factor	10	42 %	37	82 %	23	58 %	**0.005**	1.0	0.1
Diabetes	3	13 %	10	22 %	5	13 %			
Chr. Org. failure	6	25 %	29	64 %	15	38 %			
Active malignancy	1	4 %	8	18 %	5	13 %			
Immunosuppress	1	4 %	2	4 %	4	10 %			
IVDU	2	8 %	0	0 %	5	13 %			
Local risk factor	10	42 %	29	64 %	29	73 %	0.21	0.084	1.0
Rheum. joint dis.	0	0 %	4	9 %	6	15 %			
Prior SA	5	21 %	5	11 %	10	25 %			
Prosthesis	5	21 %	22	49 %	15	38 %			
Surg proc < 4 weeks	4	17 %	9	20 %	10	25 %			
Any risk factor	15	63 %	41	91 %	37	93 %	**0.046**	**0.048**	1.0

All data are presented as n (%), except Age presented as median (inner quartile range (IQR)). Significant values are highlighted in bold

*Abbreviations*: *GAS* group A streptococci, *GBS* group B streptococci, *GCGS* group C and G streptococci, *AOM* acute osteomyelitis, *PJI* prosthetic joint infection, *NJI* native joint infection, *Chr. Org.failure* chronic organ failure (according to medical records), *IVDU* active intravenous drug use, *Rheum. Joint dis* rheumatic joint disease, *SA* septic arthritis, *Surg proc < 4 weeks* surgical joint procedure performed within 4 weeks prior to admission

^*^ The *p*-values are adjusted for multiple comparisons ad modum Holm-Bonferroni

^a^ Two GCGS cases did not provide informed consent, and were excluded from the analyses

^b^ Three GAS, one GBS and one GCGS blood culture positive cases had concomitant *Staphylococcus aureus* bacteraemia. One GBS and three GCGS tissue cultures also grew coagulase negative staphylococci

**Table 2:** Clinical features, treatment and outcome

	GAS *n* = 24		GBS *n* = 45		GCGS *n* = 40 ^a^		GAS vs GBS	GAS vs GCGS	GBS vs GCGS
Symptoms							Adjusted *p*-values ^*^		
Duration days (IQR)	1	(1–5)	5	(2–13)	3	(1–9)	**0.036**	1.0	0.72
Fever	21	88 %	30	67 %	23	58 %	0.63	0.09	1.0
Severity									
SIRS	18	75 %	23	51 %	24	60 %	0.48	1.0	1.0
STSS	2	8 %	0	0 %	1	3 %	0.66	1.0	1.0
Surgical treatment									
NJI	14		12		21				
Irrigation	8	57 %	3	25 %	13	62 %			
Revision	2	14 %	2	17 %	4	19 %			
Amputation	2	14 %	0	0 %	2	10 %			
Antibiotics alone	2	14 %	7	58 %	2	10 %			
PJI	5		22		15				
Revision	5	100 %	9	41 %	7	47 %			
1-stage	0	0 %	2	9 %	1	7 %			
2-stage	0	0 %	5	23 %	3	20 %			
Amputation	0	0 %	1	5 %	1	7 %			
Antibiotics alone	0	0 %	5	23 %	3	20 %			
Treatment duration									
NJI days (IQR)	42	(32–52)	38	(28–76)	42	(34–56)			
PJI days (IQR)	90	(14–180)	137	(76–361)	91	(42–130)			
AOM days (IQR)	56	(42–56)	60	(50–116)	63	(51–81)			
Outcome									
Death <30 day	2	8 %	1	2 %	1	3 %	1.0	1.0	1.0
Sequelae	10	42 %	15	33 %	15	38 %	1.0	1.0	1.0
Amputation	2	8 %	2	4 %	3	8 %	1.0	1.0	1.0
Relapse	3	13 %	9	20 %	4	10 %	1.0	1.0	1.0
Chronic suppression	0	0 %	5	11 %	1	3 %	0.8	1.0	1.0

All data are presented as n (%), except “Duration of symptoms” and “Treatment duration” presented as median number of days (Inner Quartile Range (IQR)). Significant values are highlighted in bold

*Abbreviations*: *GAS* group A streptococci, *GBS* group B streptococci, *GCGS* group C and G streptococci, SIRS systemic inflammatory response syndrome, *STSS* streptococcal toxic shock syndrome, *NJI* native joint infection, *PJI* prosthetic joint infection, *AOM* acute osteomyelitis

^*^ The *p*-values are adjusted for multiple comparisons ad modum Holm-Bonferroni

^a^ Two GCGS cases did not provide informed consent, and were excluded from the analyses

## Results

The 15-year survey period yielded 24 GAS, 45 GBS and 42 GCGS acute Osteoarticular Infections, representing 8, 13 and 20 % of the total count of invasive episodes, respectively. Two patients with GCGS acute OAI declined participation in the study. They are represented in the cumulative incidence, but omitted from all further analyses. One GBS and three SDSE were identified based on partial sequencing of *16S rDNA* gene from the four clinical samples that were culture negative. The proportions of the different subgroups of OAI are described in Table 1.

### Annual incidence and seasonal variation

**Fig. 1:**
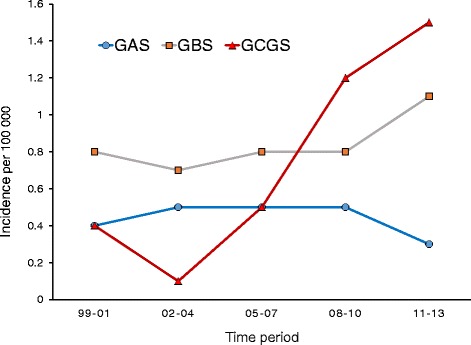
Temporal trends of osteoarticular infections caused by GAS, GBS and GCGS in western Norway 1999–2013

The cumulative incidence of GCGS OAI increased significantly from the first to the last 5-year period (IRR 5.7, *p* = 0.0003), with annual incidence peaking at 1.9/100 000 in 2013 (Fig. 1). Conversely, the cumulative incidence of GAS and GBS OAI did not display any significant trends, and remained relatively stable at 0.5/100 000 (IRR 1.27, *p* = 0.63) and 0.8/100 000 (IRR 1.77, *p* = 0.14), respectively.

All three groups exhibited seasonal variation, GAS peaking in the winter with 38 % of the cases occurring from December to February, and only 13 % during the summer-months of June through August. Differently, GBS and GCGS presented predominantly during summer (both 31 %), and less frequently in the winter (24 and 19 %, respectively).

### Demographics and comorbidity

The demographics, risk factors and disease manifestations are shown in Table 1.

**Fig. 2:**
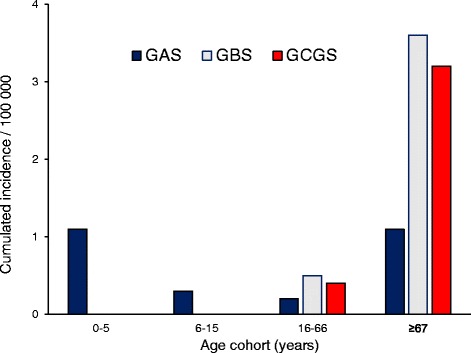
Age group related cumulative incidence of osteoarticular infections caused by GAS, GBS and GCGS

Both GBS and GCGS were significantly associated with the presence of comorbidity and risk factors; GBS clearly linked to systemic comorbidity, and GCGS predominantly associated with factors of local origin. Patients presenting with GBS or GCGS OAI were significantly older than the GAS OAI (corrected *p*-value = 0.007 and *p* = 0.049), and a male predominance was noted for GAS and GCGS. The age-difference is further delineated in Fig. 2, showing that the age-distribution for GBS and GCGS was skewed towards the elderly.

### Clinical characteristics, treatment and outcome

Details on clinical severity, treatment and outcome are provided in Table 2.

OAI caused by GAS generally presented more acutely, and were frequently admitted to hospital within 1 day of disease onset. Furthermore, they were associated with increased severity and a worse outcome as compared to OAI caused by GBS and GCGS, although the finding did not reach clinical significance. Bacteraemia was present in 58, 56 and 33 % of GAS, GBS and GCGS-cases, respectively.

Most patients were treated with a combination of surgery and antibiotics. Penicillin was prescribed in 90 % of the cases, whereas the rest were treated predominantly with Clindamycin or Cephalosporins. Between 10–20 % experienced recurring infections, the majority from relapse within 3 months after terminating antibiotic treatment. GBS was the pathogen most frequently implicated, and five patients with GBS OAI were eventually assigned to chronic suppressive antibiotic therapy. Recurrence was neither associated with antibiotic resistance patterns nor with specific *emm*/capsule-types. Although the overall mortality remained low, the morbidity was substantial. The proportion suffering from chronic sequelae ranged from 35–45 %, comprising predominantly arthritis and joint pain.

### Molecular characteristics and antimicrobial susceptibility

**Fig. 3:**
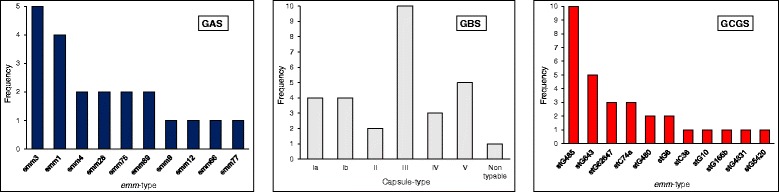
Distribution of GAS and GCGS *emm*-types and GBS capsule-types

Altogether, 21 GAS, 30 GBS and 29 GCGS isolates were available for typing, revealing a diverse microbial population (Fig. 3). The major GAS *emm*-types were *emm1* and *emm3*, and *stG485* dominated among GCGS, although neither a clonal outbreak nor a temporal trend could be inferred. Among GBS, capsule-type III and V were most frequently identified.

All isolates were sensitive to penicillin. Resistance to erythromycin was detected in 9/41 (22 %) of GBS and 4/37 (11 %) of GCGS, and resistance to clindamycin in 7/41 (17 %) of GBS but in none of the GCGS. GAS were uniformly susceptible to erythromycin and clindamycin.

## Discussion

Beta-haemolytic streptococci are an important cause of Osteoarticular infections (OAI), but their temporal trends have previously not been explored in detail. We found a highly significant increase in the GCGS OAI incidence during the 15-year survey period, the annual incidence peaking at 1.9/100 000 in 2013. To our knowledge, this has not been previously reported, although it is consistent with several epidemiological studies on invasive GCGS disease in general [11, 18, 19]. Conversely, the rates of GAS and GBS OAI remained relatively stable at 0.5/100 000 and 0.8/100 000, respectively. This contrasts the findings from a recent study, where a 3-fold increase in the incidence of GBS bone-infections in the period 1995–2012 was documented [20]. However, that study also included chronic osteomyelitis, and 76 % of the patients in their bone-infection cohort were diabetics. In line with our observations, though, they reported a stable incidence of 0.8/100 000 for GBS joint infections.

The increasing GCGS OAI incidence in our material could partly be due to increased microbial sampling or improved diagnostic procedures. However, one would expect this to be reflected also in the rates of GAS and GBS OAI, but these remained stationary. The ageing and increasingly comorbid population is probably of importance, but surprisingly did not affect the rates of GBS OAI, although GBS and GCGS both appear to preferentially target the elderly and the vulnerable patients. Acquisition of novel virulence factors enhancing invasive potential or conferring specific osteoarticular tissue tropism could be a contributing factor, and horizontal genetic transfer between streptococci has been inferred [21]. Experimental arthritis studies indicate a pivotal role for bacterial adhesins in the pathogenesis, but the knowledge on adhesins in GCGS is limited at present [22–24]. Osteoarticular infections are one of the major disease manifestations of GCGS, constituting approximately 20 % of invasive disease, and further exploration of the molecular basis for their arthritogenicity is warranted [11].

We observed several species-associated clinical characteristics. GAS OAI tended to present more acutely, to produce a more severe clinical picture, and displayed the highest propensity to cause native joint infections. GBS and GCGS affected primarily the elderly, and were significantly associated with the presence of comorbidity and risk factors; GBS were clearly linked to systemic comorbidity, whereas GCGS were predominantly associated with local risk factors. Furthermore, GBS OAI were frequently polyarticular, and comprised the majority of the recurring infections. Our findings are consistent with those from previous publications [7–10].

The antibiotic treatment was concordant with established national guidelines regarding choice of antimicrobial agent and length of treatment. The surgical intervention, however, occasionally diverged from current standards [13]. Several PJIs were treated with curative intention with antibiotics alone, particularly in the earliest time-period. This probably had an impact on the rates of recurring infections and sequelae, although, GBS have been reported to be associated with high relapse rates in prosthetic joint infections compared to other pathogens [25].

Previously, *emm4*, *emm12* and *emm28* have been associated with infective arthritis [26–28]. We found a substantial molecular diversity among our isolates, and although these three *emm*-types were represented, they did not constitute the majority (Fig. 3). On the contrary, the identified *emm*-types for both GAS and GCGS conform closely to the prevailing *emm*-types encountered in invasive disease in general during the study period [11]. Similarly, it is tempting to speculate that the OAI GBS serotypes likely reflects the most prevalent capsule types in the population, as previously documented in Norway [29].

The present study is limited by its retrospective design, increasing the risk of diagnostic misclassification. However, the microbe-specific emphasis of the study, along with a review of the local microbiological records, likely led to the inclusion of all cases of β-haemolytic streptococcal infection during the study period. Such an approach probably conforms more closely to the true disease burden than studies based on selected isolates submitted to National surveillance laboratories.

The total incidence and characteristics of OAI in our health region were unfortunately not available. Hence, a more comprehensive comparison of the clinical features of streptococcal OAI and OAI caused by other bacteria was not feasible.

Sepsis has recently been redefined, and the new classification should ideally have been applied to our sample material [30]. However, the use of the former sepsis definition based on SIRS-criteria permit a better comparison with existing literature on OAI and streptococcal disease in general. Furthermore, the retrospective design of the study hampered the categorization of the patients according to the new sepsis definition based on Sequential Organ Failure Assessment score results.

## Conclusions

We found a significantly increasing incidence of GCGS Osteoarticular infections, whereas GAS and GBS OAI did not display any temporal trends. GCGS OAI were clearly associated with the presence of comorbidity and risk factors, predominantly of focal origin. With an increasing application of therapeutic and diagnostic bone and joint procedures, the rising trend of OAI caused by GCGS is likely to continue. Sustained epidemiological attentiveness to GCGS seems warranted.

## Abbreviations

AOM: Acute osteomyelitis
GAS: Group A streptococci
GBS: Group B streptococci
GCGS: Group C and group G streptococci
GCS: Group C streptococci
GGS: Group G streptococci
IQR: Inner quartile range
IRR: Incidence rate ratio
IVDU: Intravenous drug user
NJI: Native joint infection
OAI: Osteoarticular infections
PJI: Prosthetic joint infection
SDSE: *Streptococcus dysgalactiae* subspecies *equisimilis*
SIRS: Systemic inflammatory response syndrome
STSS: Streptococcal toxic shock syndrome
